# New complementary python codes to locate Single Nucleotide Polymorphisms (SNPs) and Overlapping G-Quadruplex Sequences (G4s)

**DOI:** 10.1016/j.mex.2022.101875

**Published:** 2022-10-06

**Authors:** Mona SAAD, Marc Shebaby, Cybel Mehawej, Wissam Faour

**Affiliations:** a Gilbert and Rose-Marie Chagoury School of Medicine, Lebanese American University, Byblos, Lebanon; b School of Arts and Sciences, Lebanese American University, Byblos, Lebanon; c Department of Human Genetics, Gilbert and Rose-Marie Chagoury School of Medicine, Lebanese American University, Byblos, Lebanon

**Keywords:** G-quadruplexes (G4s), Single nucleotide polymorphisms (SNPs), Python, Overlapping G4s

## Abstract

G-quadruplexes (G4s) are non-canonical DNA and RNA secondary structures that control gene regulation. A single nucleotide polymorphism (SNP) is a small genetic variation occurring within a DNA sequence and accounting for the variabilities between individuals. While the majority of SNPs, especially those frequent in the population, are considered as benign genetic variations, few others can lead to diseases. SNPs occurring in G4 sequences were reported to modulate gene regulation. In order to find overlaps between predicted G4 sequences and SNPs located in the genomic regions, we developed two complementary computational python codes (SNP-locator and G4-overlap). The codes map a mutation to the overlapping/closest G4 sequences, based on the genetic variant name and the FASTA format of the corresponding gene. We validated these two codes on a set of 31 SNP variants occurring in cytochromes *P450* genes and podocytes-marker genes. Out of 31 SNPs, 28 were accurately located using the mentioned codes.

• SNP-locator code locates any SNP in promoters, upstream regulatory regions, exons and introns.
• The SNP-locator code requires the FASTA genomic sequence of the studied gene and the genetic variant nomenclature at the cDNA level.
• G4-overlap code maps the SNP to the overlapping or the closest G4 sequence.

## Graphical abstract

**Figure:**
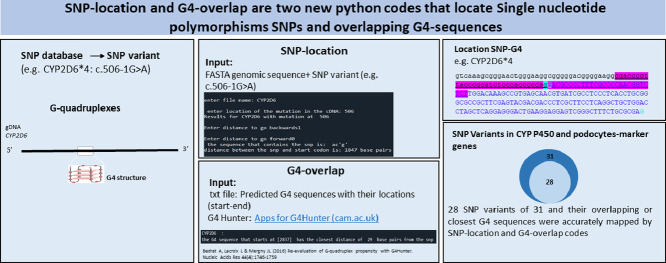

**Specifications table**

**Table:** 

Subject area:	Bioinformatics
More specific subject area:	*Genomics and molecular biology*
Name of your method:	*New complementary python codes to locate Single nucleotide polymorphism SNP and overlapping G-quadruplexes*
Name and reference of original method:	*N/A*
Resource availability	https://github.com/Marc-shebaby/SNP-G4-overlaps.git

**Method details**

## Introduction

The double helix B-DNA form can fold into other structures such as G-quadruplexes [1]. G-quadruplexes are a sort of non-canonical DNA and RNA secondary structures that form in guanine-rich regions. They have attracted the attention due to their high stability under physiological conditions [2]. Increasing evidence highlights the implication of G-quadruplexes in biological processes and in gene regulation [3,4,5]. Many algorithms were developed in order to predict the formation of these structures computationally, including recently developed G4Hunter. G4Hunter takes into account G-richness and G-skewness of the genomic sequence and gives a score as an output. This algorithm was validated experimentally and proved good accuracy in comparison with other algorithms [6].

Single nucleotide Polymorphisms (SNPs) are small genetic changes occurring within a DNA sequence. Genetic variants arising in the coding or non-coding regions may have strong influence on gene regulation and protein function and may, in some cases, be linked to clinical manifestations [7,8]. Several studies have pointed out the implication of G-quadruplexes in genomic aberrations. For example, a significant association was observed between SNPs located in G4 sequences and the expression level of the corresponding gene [9]. In addition, somatic mutations located in 5’UTR regions and altering RNA G4 stability were found to affect the regulatory function of the UTR and therefore to alter the corresponding gene expression in patients with cancer [10]. That being said, the mentioned studies relied on the manual determination of the overlap between the genetic mutations and G4 sequences, or by using an algorithm written on PERL or R. In the present work, we have developed two complementary computational codes SNP-locator and G4-overlap that were written with python. These codes can locate any SNP variant in a genomic sequence and then map it to the overlapping or the closest G4 sequence based on the G4 predictions by G4Hunter.

## Procedure

The python program (version 3.9.7) must be installed on the computer user.

SNP-locator: For the first code, a text file containing the genomic sequence of the desired gene in FASTA format and the genetic variant written as per the format set by the Human Genome Variation Society (at the cDNA level) are required. The SNP-locator code calculates the distance between the start codon and the SNP by creating two variables one for the total distance (distance) and the other for counting the base pairs in the exons (exon). Then, the lines in the text file are read until the incremented exon variable becomes equal to the distance SNP-start codon in cDNA and the total distance is given as an output.

The code takes three main inputs: The path of the folder that contains the genomic file, the name of the genomic file and the location of the mutation at the cDNA level.

If the mutation is in the intron, the code takes two additional inputs to track the location and the distance of the SNP in the intron being either ahead /forward (represented with “+”) or behind/backwards (represented with” - “) from the cDNA variant.

SNP-locator code determines the SNP by marking it with quotations and displays the distance between the SNP and the start codon. Fig. A shows an example about the application of SNP-locator code (input and output) on the SNP variant c.506-1G>A located in intron 3 of *CYP2D6* gene.

**Fig. A:**
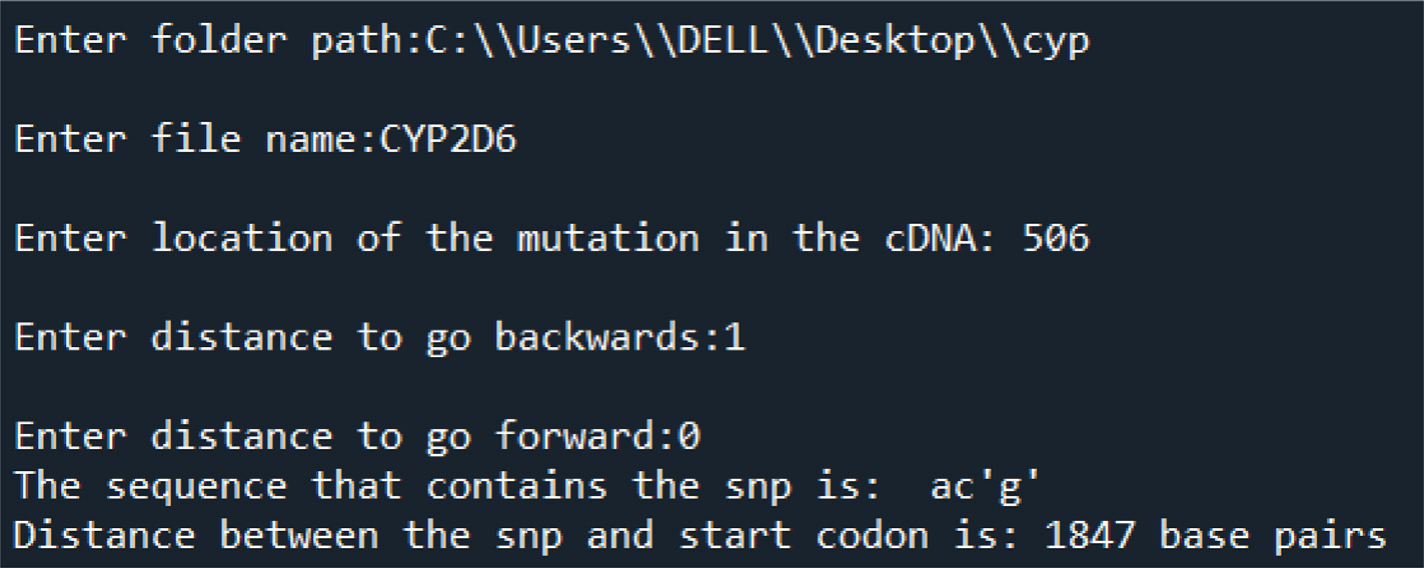
The input and output of the SNP-locator code applied to the SNP variant c.506-1G>A located in intron 3 of CYP2D6 gene.

G4-overlap: To determine the overlapping G4 sequence or the closest one to the SNP, an additional text file containing the predicted G4 sequences by G4Hunter as well as the output of the SNP-locator code are required. The G4Hunter application used is http://bioinformatics.cruk.cam.ac.uk/G4Hunter/.

The user has to save the results obtained from the first code in a text file as represented in Fig. B.

**Fig. B:**
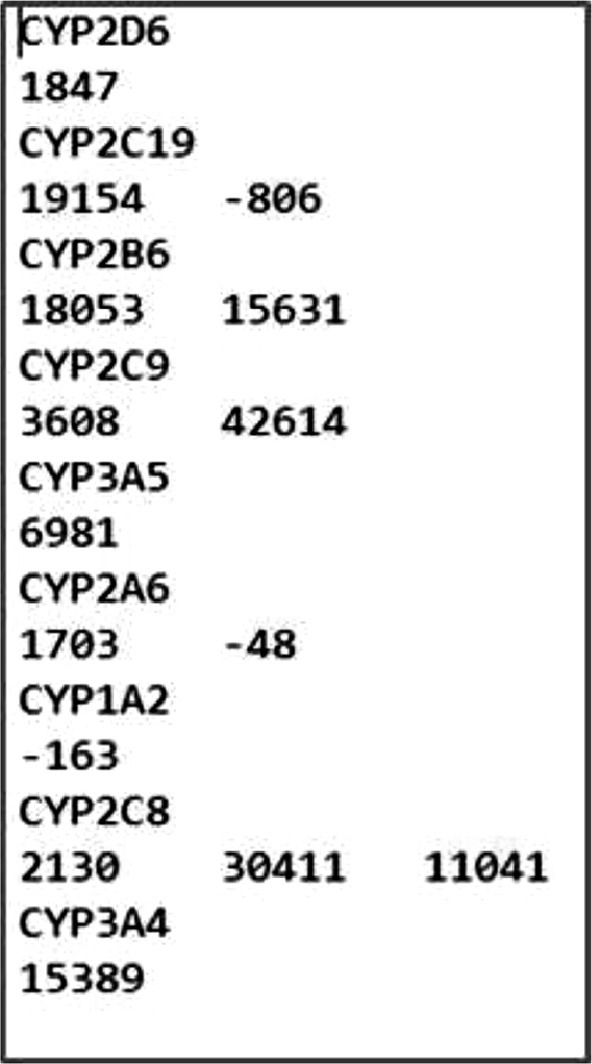
The text file including the results of the SNP-locator code for the studied genes that will be used by the G4-overlap code.

Before executing the code, the section below “seqnames” in the txt file obtained from G4Hunter has to be modified such that for every new gene the name has to be presented alone and followed by a “>” symbol (Fig. C). For every new gene, the modification should be adjusted.

**Fig. C:**
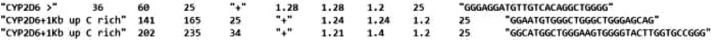
The G4 sequences predicted by the G4Hunter tool; the first occurrence of CYP2D6 in “seqnames” should be followed by “>”.

When executing the code, the user inputs the path of the folder in which the results of the first code are located and the G4-sequences exist, along with their respective file names (Fig. D).

**Fig. D:**
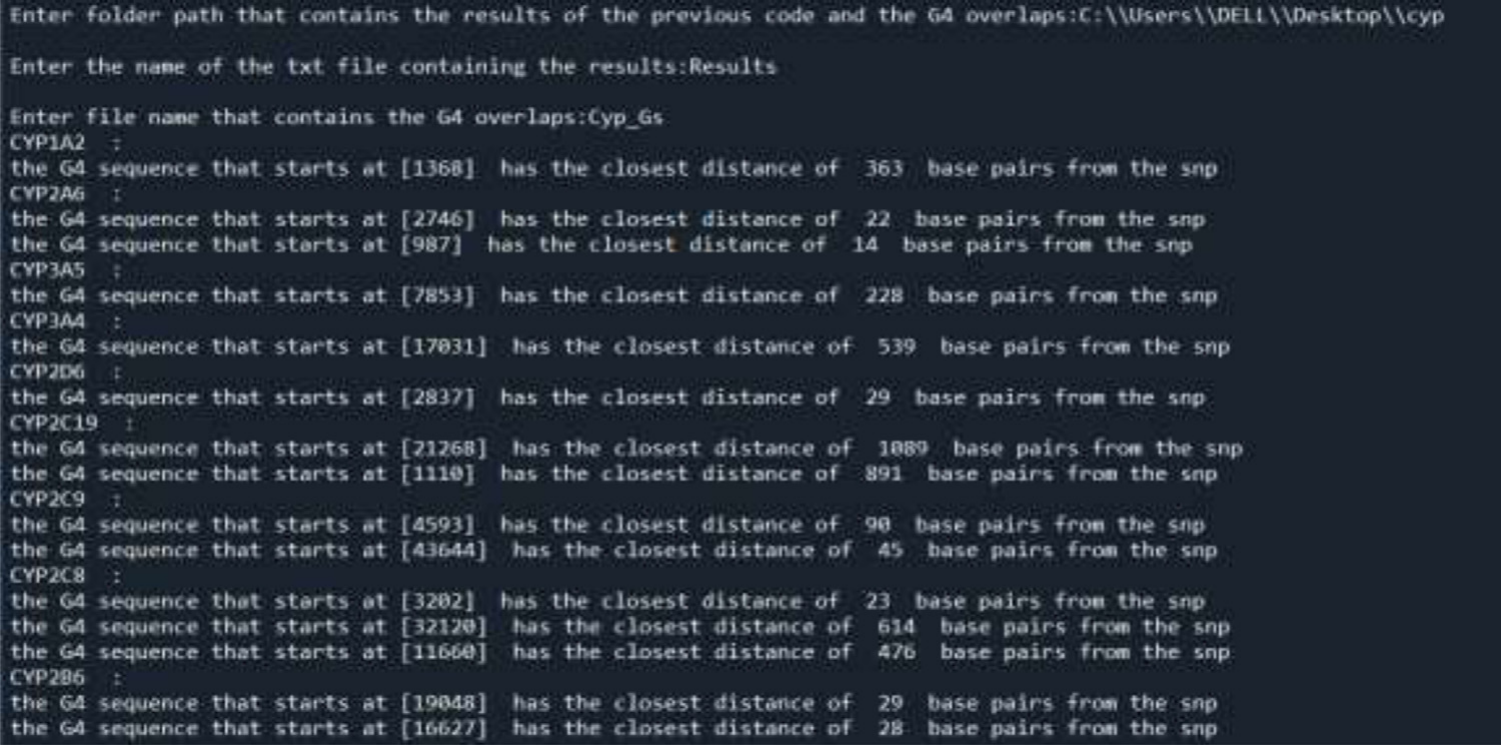
The inputs and outputs of the G4-overlap code for a group of SNP gene variants.

The distance of the overlapping or closest G4 sequence to the first codon is computed following several steps. First, the starts of the G4 sequences are subtracted from the position of the start codon in the genomic text file to obtain the distance between each G4 sequence and the start codon. Then, the distance between the SNP variant and the new computed distance for every G4 sequence is measured and stored in a list. Finally, the minimum distance from the list is extracted, and the whole process is repeated for all the SNP variants. The output given is the start and the minimum distance of the chosen G4 sequence for each gene (Fig. D).

All source code are available on GitHub (https://github.com/Marc-shebaby/SNP-G4-overlaps.git) and can be run on a personal computer.

## Method validation

We have validated these two codes on 31 genetic variants located in exons, introns and 5’ regulatory regions and then compared the location to the one determined manually using the UCSC genome browser. Out of 31 SNP variants located in the podocyte markers and *CYP450* genes, 28 were accurately mapped by these codes. Only three SNP variants were not mapped correctly due to a lack of information about the mutations and due to variations in the genomic sequences in the UCSC genome browser.

Therefore, here we propose two in-house complementary python codes that can facilitate the mapping of any mutation in any genomic location to the overlapping or the closest G4 sequence predicted by G4Hunter.

## CRediT author statement

**Wissam Faour:** Conceptualization, supervision, methodology, writing, reviewing and editing - original draft preparation; **Marc Shebaby**: Python Software methodology and validation, writing - original draft preparation; **Mona Saad**: genomic data analysis, writing - original draft preparation, Python Software methodology and validation; **Cybel Mehawej**: genomic data analysis, writing, reviewing and editing - original draft preparation.

## Declaration of Competing Interests

*Please****tick****the appropriate statement below (please do not delete either statement) and declare any financial interests/personal relationships which may affect your work in the box below*.

☒ The authors declare that they have no known competing financial interests or personal relationships that could have appeared to influence the work reported in this paper.

☐ The authors declare the following financial interests/personal relationships which may be considered as potential competing interests: *Please declare any financial interests/personal relationships which may be considered as potential competing interests here*.

## Appendix Supplementary materials

Image, application 1

Image, application 2

Image, application 3
